# Modular Fabrication of Microfluidic Graphene FET for Nucleic Acids Biosensing

**DOI:** 10.1002/advs.202401796

**Published:** 2024-07-23

**Authors:** Qiongdi Zhang, Yuxuan Hao, Tonghua Zeng, Weiliang Shu, Pan Xue, Yang Li, Chi Huang, Liwei Ouyang, Xuming Zou, Zhen Zhao, Jiahong Wang, Xue‐Feng Yu, Wenhua Zhou

**Affiliations:** ^1^ Shenzhen Institute of Advanced Technology Chinese Academy of Sciences Shenzhen 518055 China; ^2^ Southern University of Science and Technology Shenzhen 518055 China; ^3^ Key Laboratory for Micro/Nano Optoelectronic Devices of Ministry of Education and Hunan Provincial Key Laboratory of Low‐Dimensional Structural Physics and Devices School of Physics and Electronics Hunan University Changsha 410082 China; ^4^ The Key Laboratory of Biomedical Imaging Science and System Chinese Academy of Sciences Shenzhen 518055 China

**Keywords:** biosensor, CRISPR/Cas12a, graphene field‐effect transistor, microfluidic, modular fabrication

## Abstract

Graphene field‐effect transistors (GFETs) are widely used in biosensing due to their excellent properties in biomolecular signal amplification, exhibiting great potential for high‐sensitivity and point‐of‐care testing in clinical diagnosis. However, difficulties in complicated fabrication steps are the main limitations for the further studies and applications of GFETs. In this study, a modular fabrication technique is introduced to construct microfluidic GFET biosensors within 3 independent steps. The low‐melting metal electrodes and intricate flow channels are incorporated to maintain the structural integrity of graphene and facilitate subsequent sensing operations. The as‐fabricated GFET biosensor demonstrates excellent long‐term stability, and performs effectively in various ion environments. It also exhibits high sensitivity and selectivity for detecting single‐stranded nucleic acids at a 10 fm concentration. Furthermore, when combined with the CRISPR/Cas12a system, it facilitates amplification‐free and rapid detection of nucleic acids at a concentration of 1 fm. Thus, it is believed that this modular‐fabricated microfluidic GFET may shed light on further development of FET‐based biosensors in various applications.

## Graphical abstract

A modular fabrication strategy of microfluidic graphene FET biosensor for nucleic acids detection is designed based on PDMS technique and low‐melting metal electrodes. The device is fabricated through three simple and independent steps within 1.5 hours. The as‐fabricated biosensor exhibits long‐term stability and good performances in various ionic environments, and enables reliable detection of nucleic acids down to fm range.

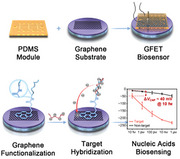

## 1 Introduction

Over the past twenty years, field‐effect transistor (FET) has undergone an intense development in biosensing, due to the excellent signal amplification capability which greatly improved detection limits.^[^ ^1^, ^2^ ^]^ Among the abundant channel materials, graphene is an emerging candidate for its unique properties including large surface‐to‐volume ratio, high carrier mobility, capability of large‐area growth and good stability under ambient conditions,^[^ ^3^, ^4^, ^5^, ^6^ ^]^ making it suitable for the surface functionalization and biosensing. Therefore, graphene FET (GFET) is regarded as a promising platform for all‐round biomarkers sensing, including proteins,^[^ ^7^, ^8^ ^]^ metabolites,^[^ ^9^, ^10^ ^]^ and nucleic acids.^[^ ^11^, ^12^ ^]^ Typical GFET‐based biosensors are constructed with multiple steps of Micro‐Electro‐Mechanical System (MEMS) standard fabrication, including repeated lithographic techniques, etching, metallization, dielectric deposition, lift‐off, etc., in which sophisticated facilities and strict conditions are required.^[^ ^13^, ^14^, ^15^ ^]^ The complicated and expensive process line hinders the wide research and applications of GFET. Additionally, the existence of the photoresist and chemical residues at various fabrication stages may lead to imperfect surfaces and material breakages, which impact the performance of the devices.^[^ ^13^ ^]^ Furthermore, since even a solitary error in the process can lead to the failure of the entire device,^[^ ^13^, ^16^ ^]^ it is essential to streamline the fabrication procedure with fewer steps to enhance the yield of GFET biosensors, making it critical for their real‐world applications.

Microfluidics is a multidisciplinary technology that simplifies fabrication strategy and combines sensing devices with liquid manipulations in micro‐volumes onto a single chip.^[^ ^17^, ^18^ ^]^ By integrating biosensors with microfluidics, it is possible to create lab‐on‐a‐chip systems that can handle full sample treatment and analysis, while also miniaturizing the sensing platform, enabling portability for point‐of‐care testing in future clinical diagnosis.^[^ ^19^, ^20^ ^]^ Additionally, microfluidic components can provide confined chambers or channels to form independent modules, such as pumps,^[^ ^21^ ^]^ valves,^[^ ^22^ ^]^ heaters,^[^ ^23^ ^]^ and electrodes,^[^ ^24^, ^25^ ^]^ which can be easily transferred to different substrates and assembled with various functional units. Overall, microfluidic biosensors have diverse applications in biomarker sensing such as enzymes,^[^ ^26^ ^]^ antibodies,^[^ ^27^ ^]^ and nucleic acids.^[^ ^28^ ^]^ Various detection approaches, including electrochemical,^[^ ^29^ ^]^ optical,^[^ ^30^ ^]^ magnetic,^[^ ^31^ ^]^ and electronic methods,^[^ ^32^ ^]^ have greatly broadened the biosensor's capabilities. However, current microfluidic graphene FET biosensors are typically constructed separately and then integrated with long flow channels, resulting in a time‐consuming process and low yield.^[^ ^13^, ^14^ ^]^

The deformability of low‐melting metals enables direct injection into microfluidic channels at a suitable temperature, and solidify into electrodes.^[^ ^33^, ^34^, ^35^ ^]^ This integration eliminates the requirement for an encapsulation layer for the metallic electrodes, while also protect the electrodes from damages or short circuits caused by the liquid sensing environment.^[^ ^33^, ^34^, ^35^ ^]^ Consequently, the integration of low‐melting metal electrodes with microfluidics could dramatically simplify the fabrication process of FET devices in a modular manner, while maintaining both good electrical conductivity as well as the cleanliness and integrity of substrate surface.

In this work, we proposed a simple, modular, and contamination‐free fabrication technique to construct low‐melting metal‐based microfluidic GFET biosensors. Our approach only took 3 independent and facile steps compared to traditional complicated fabrication processing that almost had 13 sequentially dependent steps (Figure S1, Supporting Information). The as‐fabricated GFET biosensor demonstrated good stabilities and sensing performances in different liquid environments. Moreover, this modular‐fabricated biosensor showed high sensitivity and selectivity to single‐stranded nucleic acids detection at a concentration down to 10 fm, which is comparable with current industry‐standard fabricated DNA sensors. Furthermore, it illustrated the possibility to be combined with the CRISPR/Cas12a system for amplification‐free and rapid nucleic acid detection. This strategy offers the possibility of adapting other nanomaterials, beyond graphene, in FET architectures. Furthermore, microfluidic FET based on low‐melting or liquid metals demonstrates a wide variety of applications in soft, flexible, and wearable electronics, showing great potentials towards low cost, high maturity and customization electronic sensor devices.

## 2 Results and Discussion

**Figure 1:**
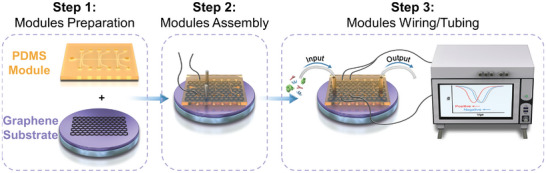
Schematic illustration of the microfluidic GFET biosensor fabrication processing.

The fabrication of microfluidic GFET biosensor consisted of three independent steps, briefly, the preparation of patterned polydimethylsiloxane (PDMS) module, the assembly of graphene‐PDMS module, and the wiring/tubing of modules (**Figure** **1**). In particular, the fabrication processing was started by negatively transferring PDMS module from a patterned SU‐8 mold, which included two architectures of microfluidic channel: one was for the low‐melting metal electrodes, the other was for the fluids. Then, the PDMS module was integrated and immobilized on the top of the graphene substrate under an external pressure by using two plates. And the source drain electrodes were formed by low‐melting metals that were injected into the microfluidic channels at a certain temperature and then solidified into electrodes. Finally, the electrical wires and fluidic tubes were connected to the exterior instruments, and the whole setup was prepared for experiment (Figure S2, Supporting Information). This sequentially independent 3‐step modular fabrication method would greatly simplify microfluidic GFET device fabrication and increase device yield.

**Figure 2:**
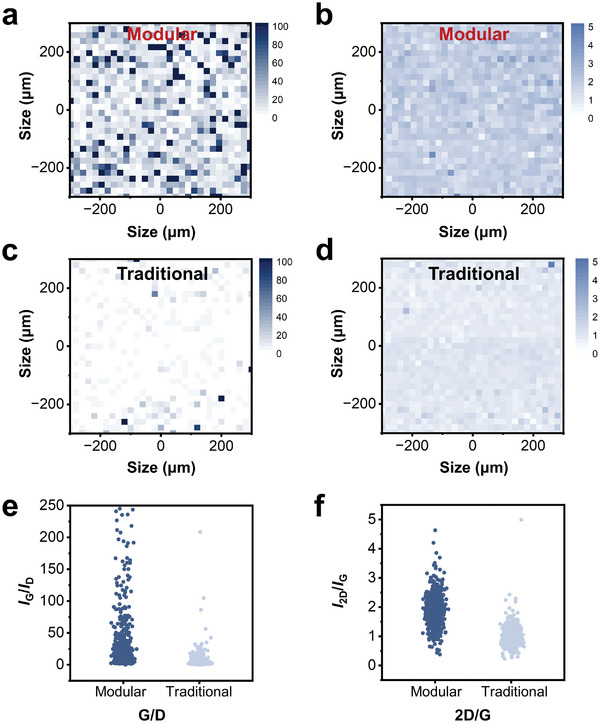
Characterizations of the graphene surface quality. a,b) Raman spectra mapping of (a) G/D and (b) 2D/G intensity ratios of graphene obtained by the modular fabrication. c,d) Raman spectra mapping of (c) G/D and (d) 2D/G intensity ratios of graphene obtained by the traditional fabrication. e,f) Comparisons of intensity ratios of (e) G/D and (f) 2D/G peaks between the two fabrication methods.

Since no photoresist and other chemical residues were introduced onto the graphene substrate in this method, an important advantage in this fabrication process is contamination‐free. Typically, in the Raman spectra of the pristine graphene, there are two major peaks. The G peak at ≈ 1580 cm^−1^ that corresponds to the lattice vibration mode, and the 2D peak at ≈ 2700 cm^−1^ that is attributed to the second‐order Raman scattering and is often used for the determination of the numbers of graphene layer.^[^ ^36^ ^]^ Other peaks such as the D peak at ≈ 1350 cm^−1^ also appears at the graphene edge and is usually related to the presence of defects in the graphene.^[^ ^36^, ^37^ ^]^ Thus, we characterized and compared the quality and integrity of graphene surfaces treated with the modular fabrication and the traditional one respectively by Raman spectroscopy. **Figure** **2**a–d displays the Raman mapping of final graphene obtained by two different methods. In the 300 × 300 µm^2^ areas, the intensity ratios of both the G/D peak (Figure 2e) and the 2D/G peak (Figure 2f) of graphene surface treated with our approach were higher than that treated with the traditional one. The results indicated that our method retained much more single layers of graphene and caused less disorders in graphene surface, thereby the modular fabrication approach preserved higher quality and integrity of graphene surface than the traditional repeated lift‐off fabrication strategy.

Besides surface cleanliness and integrity, we also characterized the contact between the graphene surface and the low‐melting metal electrodes in liquid phase, as shown in the output characteristics in Figure S3 (Supporting Information). The currents increased almost linearly as the drain voltage increased, indicating the good Ohmic contacts between the graphene surface and the electrodes.^[^ ^7^ ^]^ Therefore, high quality and integrity of the graphene surface in liquid environments were proved, demonstrating that the modular fabrication approach was facile, contamination‐free, and harmless to the graphene surface.

**Figure 3:**
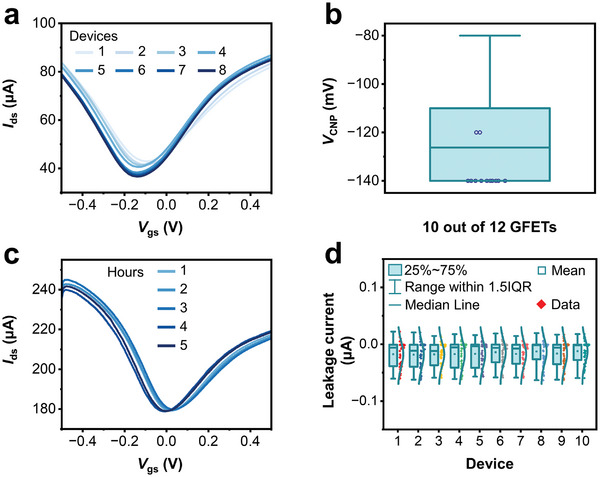
Reliability of the microfluidic GFET biosensor. a) Charge transfer characteristics of 8 individual GFETs in 10× PBS solution. b) *V*_CNP_ of 12 individual GFETs in 10× PBS solution. c) Transfer curves of the GFET in 1× PBS solution measured with respect to time. d) Leakage current of 10 individual GFETs in 1× PBS solution.

Given the good quality of the graphene surface after the proposed processing, we further investigated the stability and reproducibility of the as‐fabricated microfluidic GFET biosensors under physiological conditions. **Figure** **3**a shows the charge transfer characteristics of 8 individual GFETs in static 10× phosphate buffer saline (PBS) solution, where the drain voltage *V*_ds_ was fixed to 0.1 V and the sweep step was 20 mV. By altering the gate voltage *V*_gs_ from −0.5 V to 0.5 V, all 8 GFETs exhibited the typical ambipolar property of graphene, of which the type of charge carriers in graphene could be modulated continuously from holes to electrons.^[^ ^38^ ^]^ A statistic of the charge neutrality point voltage (*V*_CNP_) was counted and shown in Figure 3b. We measured 12 devices, and 10 out of 12 had identical *V*_CNP_ values of −140 mV, indicating good reproducibility of the as‐fabricated microfluidic GFET biosensors.

Because the graphene modification and biological detection are usually carried out in liquid conditions, long‐term stability of the microfluidic GFET biosensors in contact with physiological solutions is a pre‐requisite for robust biosensing. We then evaluated the stability of GFETs by measuring the transfer curves after continuous incubation with 1× PBS. As shown in Figure 3c, the *V*_CNP_ of a randomly selected device only presented a slight change after 5 hours in buffer solution, indicating well‐maintained electronic characteristics of the GFET. In addition, to investigate the encapsulation reliability of the device, we recorded the leakage currents of 10 GFETs under different gate voltages and zero drain voltage (Figure 3d; Figure S4, Supporting Information). The absolute values of these leakage currents were always below 100 nA, which was negligible and thus demonstrated the good electrode encapsulation of the microfluidic GFET biosensors.^[^ ^39^ ^]^

**Figure 4:**
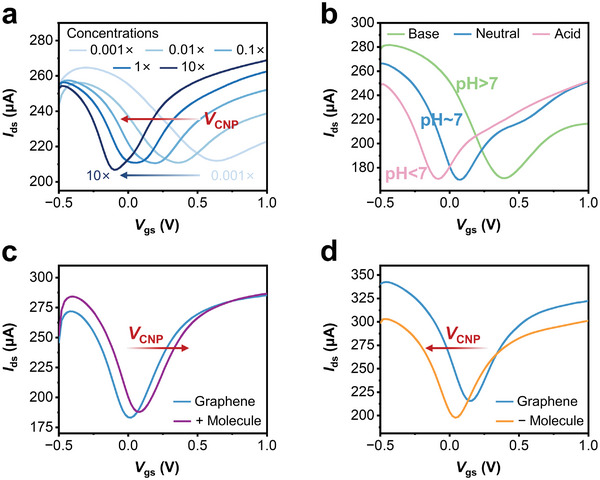
Sensing performances of the microfluidic GFET biosensors in different aqueous solvents. a) Charge transfer characteristics of GFET as a function of PBS concentration with channel length of 50 µm. b) Charge transfer characteristics of GFET as a function of pH value. c) Charge transfer characteristics of GFET with the addition of positively charged biomolecule. d) Charge transfer characteristics of GFET with the addition of negatively charged biomolecule.

Subsequently, the sensing performances of the microfluidic GFET biosensors in various aqueous solvents were investigated, including different salt concentrations, pH values, and charged biomolecules. **Figure** **4**a displays the transfer curves of the device against PBS buffer concentrations, in which a negative correlation between *V*_CNP_ and PBS concentration was observed (Figure S5, Supporting Information). This transfer curve shift is mainly attributed to the potential drop across the electrical double layer (EDL) in electrolyte/graphene interfaces.^[^ ^40^ ^]^ Generally, the inside layer of EDL contains the opposite charges to the graphene, while the outer is a diffuse layer that consists of both ions and counterions. The length of the EDL can be defined as the Debye length (*λ*_D_) as represented by Equation 1:

(1) $$\;\lambda_{D}=\;\sqrt{\frac{\varepsilon_{0}\varepsilon_{r}k_{B}T}{2N_{A}e^{2}I}}$$

where *ε*_0_ is the vacuum permittivity, *ε*_r_ is the relative permittivity or the dielectric constant of the electrolyte in this case, *k*_b_ is the Boltzmann's constant, *T* is the absolute temperature, *N*_A_ is the Avogadro's number, *e* is the elementary charge, and *I* is the ionic strength of the electrolyte.

According to the Equation 1, the Debye length (*λ*_D_) is highly influenced by the ionic strength.^[^ ^41^, ^42^, ^43^ ^]^ Typically, in PBS buffer system, studies have proved that the Debye lengths of 1× PBS, 0.1× PBS, 0.01× PBS and 0.001× PBS were estimated at about 0.7 nm, 2.3 nm, 7.2 nm and 23 nm, respectively.^[^ ^44^ ^]^ That means the higher the ionic concentration, the higher the ionic strength is, which in turn decreases the Debye length. Meanwhile, the decrease of the Debye length leads to the increase of the EDL capacitance, which in turn reduces the surface potential for a given surface charge, i.e., a left shift of the *V*_CNP_ in our experiment. In addition, the increase of the ionic strength gives rise to the increase of the doping effect, also known as the direct charge transfer to the graphene, which causes the left shift of the *V*_CNP_ as well.^[^ ^40^ ^]^ Devices with a wider channel length (500 µm) also demonstrated the same shift in gate voltage as the PBS concentration increased (Figure S6, Supporting Information). All these results proved good sensing ability of the microfluidic GFET biosensor to different salt concentrations.

Because pH value is usually an important factor for biological reactions, the responses of the microfluidic GFET biosensors to different pH environments were also examined. We prepared three solutions with pH values of 3.5 (10 mm citrate buffer), 7.3 (10 mm PBS buffer) and 8.6 (10 mm borate buffer). The *V*_CNP_ shifted towards positive and negative voltage when exposed to basic and acidic buffer respectively, as shown in Figure 4b and Figure S7 (Supporting Information). This could be considered as the adsorption of hydroxide ions in basic solution onto graphene that would cause an excess of holes in the graphene surface, i.e., a p‐type doping of graphene. Conversely, hydroxonium ions in acidic solution would lead to an excess of electrons in the graphene surface, forming a n‐type doping.^[^ ^45^, ^46^ ^]^

In addition to pH value sensing, we also investigated detecting performance of differently charged biomolecules adsorbed onto graphene surface. Poly‐L‐lysine (PLL) and bovine serum albumin (BSA) were dissolved in 1× PBS buffer at pH 7.4, separately. Thus, PLL would become positively charged in this PBS solution via protonation of the carboxyl groups.^[^ ^47^ ^]^ Accordingly, BSA would become negatively charged via deprotonation of protons from the ammonium groups.^[^ ^48^ ^]^ Figure 4c,d show the transfer curves of the microfluidic GFET biosensors before and after PLL and BSA adsorption onto the graphene surface respectively. We observed that the positively charged biomolecules induced positive shift of the *V*_CNP_, exhibiting p‐doped transport behavior (Figure 4c), while the negative charges exhibited n‐doped transport behavior (Figure 4d). These circumstances of carriers in graphene might be considered as a result of the doping effect, which means the direct charge transfer from biomolecules to graphene.^[^ ^49^ ^]^

**Figure 5:**
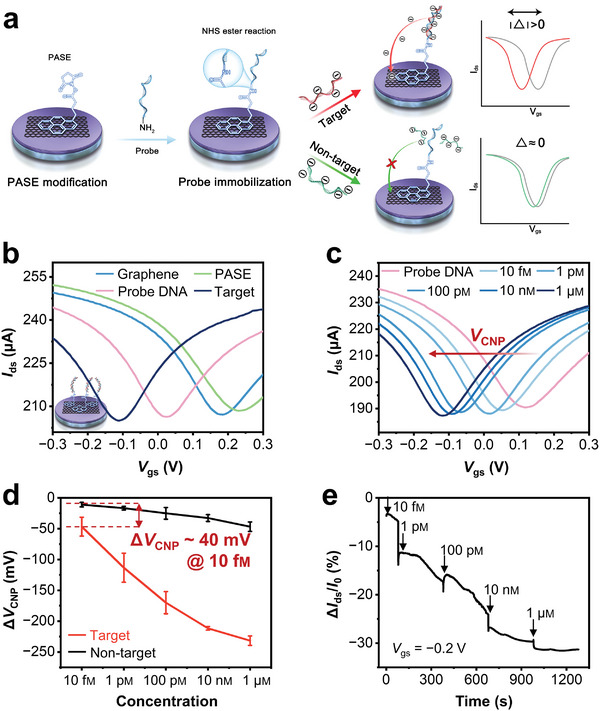
Nucleic acids hybridization sensing by the microfluidic GFET biosensors. a) Sensing mechanism of GFET for complementary target and its negative control. The graphene surface was first modified by PASE through π‐π stacking, then amine‐modified probe DNAs was immobilized onto the surface by covalent amide bonds. If the target was complementary to the probe, the hybridization would introduce an obvious shift of electronic signals, otherwise, the shift was negligible. b) Charge transfer characteristics of step‐by‐step surface modification for complementary target detection. c) Charge transfer characteristics of GFET for the hybridization sensing at different target concentrations, added from 10 fm to 1 µm in sequence. The drain voltage *V*_ds_ = 0.1 V, the gate voltage *V*_gs_ varied from −0.3 V to 0.3 V, and the sweep step = 5 mV. d) Comparison of the *V*_CNP_ shift for complementary target and non‐target at different concentrations. The baseline represents the *V*_CNP_ value after immobilized by probe DNA, *n* = 3, mean ± std. e) Real‐time measurement of nucleic acids hybridization, where *I*_0_ indicates the primary current, *I* is the real‐time current that was collected every one second, Δ*I*_ds_ is the difference of *I* and *I*_0_. The gate voltage was fixed at *V*_gs_ = −0.2 V, and the drain voltage was maintained at *V*_ds_ = 0.1 V.

Given the good reproducibility, stability and sensing performance of the prepared microfluidic GFET biosensor, we further tested its potential in target nucleic acids detection. **Figure** **5**a illustrates the sensing mechanism of the microfluidic GFET biosensor for complementary target and its negative control. The graphene surface was firstly modified by linker molecule, i.e., 1‐pyrenebutanoic acid succinimidyl ester (PASE). PASE is rich in pyrene groups so that it could be bound to graphene through π‐π stacking, while the succinimide group in the other end permits the immobilization of amine‐modified probe DNAs through a covalent bonding.^[^ ^12^, ^32^ ^]^ After PASE functionalization, the probe DNAs were immobilized onto the graphene surface by covalent bonding between 5′‐NH_2_ of the probe and succinimide group of the PASE via a NHS ester reaction. Both modification steps were conducted inside the PDMS module with the help of pump and syringe to inject corresponding solutions. Figures S8 and S9 (Supporting Information) show the stabilities of GFETs after PASE functionalization and probe DNA immobilization, respectively. Even incubated in buffer for 5 hours after surface modification, the device exhibited good stability and performances. All oligonucleotide sequences used in this work and modification protocols were detailed in Table S1 (Supporting Information) and Experimental Section, respectively.

Figure 5b and Figure S10 (Supporting Information) display the charge transfer characteristics of step‐by‐step surface modification for complementary target and non‐target detection, respectively. As shown in Figure 5b, the *V*_CNP_ shifted to positive gate voltage after PASE functionalization, which could be explained by the charge transfer between pyrene group and graphene through π‐π stacking that intensifies p‐type doping effect on graphene.^[^ ^32^, ^50^ ^]^ The *V*_CNP_ then shifted to negative gate voltage after probe DNA immobilization, which is due to the n‐type doping effect on graphene that the nucleotides are rich in electrons and could act as electron donors.^[^ ^12^, ^38^, ^51^ ^]^ These results confirmed good performances of the microfluidic GFET biosensor during surface modifications. Then, we carried out the detection to a synthetic target (a 29‐mer RNA oligo derived from the conserved nucleocapsid gene of SARS‐CoV‐2 genomic sequence fully complementary to the probe DNA) and its negative control (a single nucleotide mutant of the synthetic target). Since double‐stranded DNA‐RNA hybrids possess much more electrons, a more negative shift of the *V*_CNP_ was imposed after introducing and incubating with the complementary targets (Figure 5b). In contrast, the negative control resulted in little movement of the *V*_CNP_ because the non‐target cannot be specifically hybridized to the probe DNA (Figure S10, Supporting Information). Such distinguishing shift of the *V*_CNP_ between target and non‐target demonstrated good sensing capability of our microfluidic GFET biosensor.

To further study the sensitivity and specificity of the microfluidic GFET biosensor, we investigated the limit of detection by altering target concentration, as shown in Figure 5c. The *V*_CNP_ shifted gradually to the negative voltage direction as the concentration of fully complementary target increased from 10 fm to 1 µm in sequence, which indicated the progress of complementary targets specifically hybridized to the probe DNAs, thus resulting in a much more n‐type doping effect of graphene.^[^ ^12^, ^51^, ^52^, ^53^ ^]^ Figure 5d reveals the specific detection between complementary target and non‐target with one nucleotide mismatch at different concentrations. For the non‐target, no significant shift was observed at 10 fm concentration, whereas for the same concentration of complementary targets, the *V*_CNP_ shift was obviously observed with about 50 mV. The results demonstrated that our microfluidic GFET biosensor had high sensitivity and selectivity to single‐stranded nucleic acids detection in the fm range (Table S2, Supporting Information), which also could be comparable with current industry‐standard fabricated DNA sensors.^[^ ^11^, ^32^ ^]^

Real‐time detection is important in most point‐of‐care clinical diagnosis applications, we thus recorded the real‐time responses of the microfluidic GFET biosensor to the hybridization of complementary target, as shown in Figure 5e. The real‐time Δ*I*_ds_/*I*_0_ response displayed a stepwise current increase upon hybridizing of specific target with increasing concentration. Moreover, the device was able to effectively detect target in a short time (within 5 min) and achieved a limit of detection down to 10 fm. This result demonstrated great potentials of our microfluidic GFET biosensor for point‐of‐care testing in the future.

**Figure 6:**
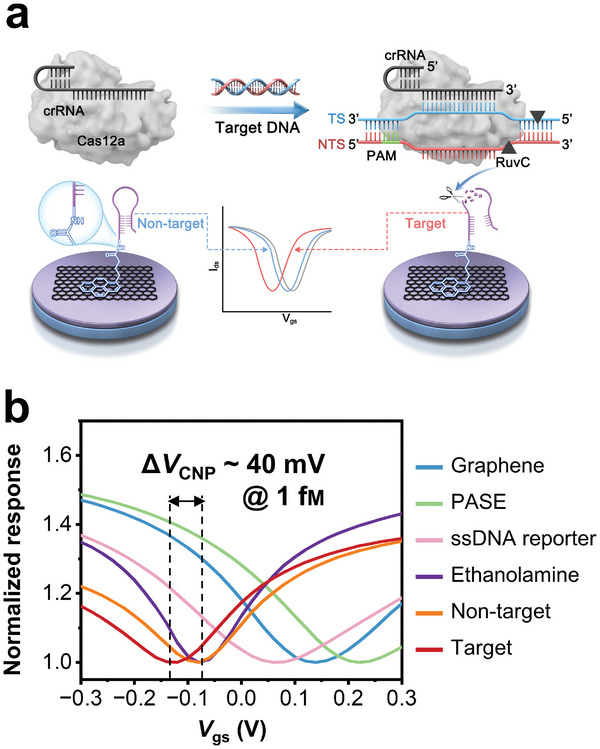
Nucleic acids detection by combining CRISPR/Cas12a system and the microfluidic GFET biosensor. a) Sensing mechanism of the coupled CRISPR/Cas12a‐GFET system. The ssDNA reporter was immobilized onto the graphene surface via PASE linker molecule as mentioned above. b) Normalized responses of CRISPR/Cas12a‐GFET of each functionalization process and toward the complementary target and its negative control.

Since CRISPR/Cas12a system has been emerging as a revolutionary diagnostic tool due to its high‐sensitivity, high‐speed and amplification‐free in nucleic acid detection,^[^ ^54^, ^55^, ^56^, ^57^, ^58^, ^59^ ^]^ we performed preliminary investigation combining our microfluidic GFET biosensor with CRISPR/Cas12a system. As shown in **Figure** **6**a, the detection system was established by taking the advantage of the collateral activity (trans‐cleavage) of Cas12a that once activated by target complementary to crRNA, resulting in a *V*_CNP_ shift of the signal due to the nonspecific degradation of single‐stranded DNA (ssDNA) reporters immobilized on the graphene surface.^[^ ^54^, ^55^ ^]^ As shown in Figure 6b (rose curve), a left shift of the *V*_CNP_ was observed after the ssDNA reporter immobilization, suggesting the transferred electrons of the graphene surface from the phosphate backbone of ssDNA reporters.^[^ ^54^ ^]^ Once CRISPR/Cas12a complex was introduced to the graphene surface in the presence of 1 fm double‐stranded target DNA (between 11703–11761 of the conserved hexon gene of human adenovirus type 7), a left shift of the *V*_CNP_ was observed (Figure 6b, red curve). Whereas, for the non‐target (a double‐stranded DNA between 12994–13052 of human adenovirus type 7), there was no shift of signal observed (Figure 6b, orange curve). The statistical analysis shown in Figure S11 (Supporting Information) presented a high selectivity of the biosensor to distinguish the complementary target from non‐target. Moreover, the specific recognition of the sequence of human adenovirus type 7 (HAdV‐7) among several viral species based on this CRISPR/Cas12a‐GFET biosensor was also investigated, corresponding results in Figure S12 (Supporting Information) further confirmed its superior specificity. Overall, the results fully demonstrated the combination potential of our proposed fabricated microfluidic GFET biosensor with the CRISPR/Cas12a system despite relative high target concentration. In the future studies, optimizations regarding the reporter designs and detecting strategies will be performed to further improve the limit of detection of our microfluidic GFET biosensors in combination with CRISPR systems.

## 3 Conclusion

We developed a simple, modular, and contamination‐free fabrication strategy to construct a low‐melting metal‐based microfluidic GFET biosensor. Compared to the traditional complicated fabrication processing, our method had only three steps. The as‐fabricated GFET biosensor demonstrated excellent long‐term stability, and performed effectively in various liquid phase environments. Additionally, the biosensor offered good sensing performances for different ionic strength solutions, pH value environments, as well as differently charged biomolecules. Moreover, it demonstrated a high sensitivity and selectivity of nucleic acids hybridization detection at a concentration down to 10 fm. Meanwhile, it enabled a combination with CRISPR/Cas12a system for amplification‐free and rapid nucleic acid detection. Taking advantages from the current A4‐size growth and transfer‐free technologies of continuous graphene films, for example, the super graphene‐skinned materials,^[^ ^60^, ^61^ ^]^ as well as the mature PDMS array processing technique, the proposed modular fabrication strategy would enable hundreds of tests to various biomarkers simultaneously. Therefore, we anticipate that this modular‐fabricated approach would extend the availability of GFET biosensors to more interdisciplinary applications and enable more rapid and reliable adaptation to various novel FET device architectures.

## 4 Experimental Section

### Materials and Reagents

Monolayer graphene films of 5 cm × 10 cm were ordered from Shenzhen SixCarbon Technology. SU‐8 3050 photoresist and Dow Corning Sylgard 184 Silicone Elastomer Kit were purchased from Suzhou CChip scientific instrument Co., Ltd. 1‐pyrenebutanoic acid succinimidyl ester (PASE), ethanolamine, and borate buffer were purchased from Aladdin (Shanghai, China). Citrate buffer was purchased from Bidepharm (Shanghai, China). Poly‐l‐lysine (PLL) and bovine serum albumin (BSA) were purchased from Beyotime (Shanghai, China). 10× phosphate‐buffered saline (PBS) solution (1.37 m NaCl, 81 mm Na_2_HPO_4_, 26.83 mm KCl and 17.6 mm KH_2_PO_4_ at pH 7.4) and ammonium persulfate were purchased from Sangon Biotech (Shanghai) Co., Ltd. 5′‐amine‐modified probe DNA oligo, 29‐mer complementary RNA target, 90‐mer complementary RNA target and one‐base mismatched non‐target were synthesized and provided by IGE Biotech (Guangzhou, China). Lachnospiraceae bacterium ND2006 (LbCas12a) was purchased from NEB. 5′‐amine‐modified ssDNA reporter, crRNA, complementary dsDNA target of HAdV‐7 and its non‐target were synthesized and provided by Generay (Shanghai, China). Monkeypox virus (MPXV) pseudoviral genomic sequence was ordered from Yeasen Biotechnology (Shanghai) Co., Ltd. Hepatitis B (HBV), Human Papillomavirus Type 16 (HPV‐16) and HPV‐18 pseudoviral genomic sequences were purchased from Guangzhou BDS Biological Technology.

### Graphene Substrate Preparation

The single layer graphene purchased from Shenzhen SixCarbon Technology was synthesized on Cu foil by chemical vapor deposition, and was protected by a polymethyl methacrylate (PMMA) thin layer. To transfer the graphene onto the 300 nm SiO_2_/Si substrate, a wetting method was adopted. Briefly, the PMMA‐coated graphene/Cu was first cut in the size of 0.5 cm × 1.5 cm, and immersed into a 10% ammonium persulfate solution for about 2 h to etch copper foil. Then the PMMA‐coated graphene was rinse by deionized (DI) water repeatedly, and was transferred onto a 2‐inch SiO_2_/Si substrate. The following steps were drying the substrate for 30 min and baking at 90 °C 30 min then 130 °C 30 min, in a nitrogen atmosphere. Finally, the PMMA layer was removed by soaking in acetone solution for about 1 h, and followed by rinsing with isopropanol, ethanol and DI water respectively. The final graphene substrate was done by annealing at 130 °C for 30 min in nitrogen atmosphere.

### PDMS Module Preparation

A bare 4‐inch silicon wafer was firstly spin‐coated with SU‐8 3050 photoresist (20 s at 500 rpm then 30 s at 3000 rpm) and soft‐baked at 65 °C for 5 min, then 95 °C for 25 min. Once cooled down, the SU‐8 wafer was patterned using UV‐exposure, and followed by a post exposure bake at 65 °C for 5 min then 95 °C for 3 min. After cooling down, the SU‐8 mold was developed in SU‐8 developer and hard baked at 150 °C for 30 min. The patterns were then negatively transferred to PDMS (ratio of A:B = 10:1), cured in an oven for about 20 min at 80 °C.

### Microfluidic GFET Biosensor Preparation

The as‐prepared graphene substrate and PDMS module were bonded together under an external pressure by using two customized plates (aluminum and polycarbonate plates), and were fixed by employing four screws (Figure S1, Supporting Information). The low‐melting metal was injected into the microfluidic channels at 110 °C on a hot plate then solidified into source and drain electrodes when come back to the room temperature. The exterior connection wires were linked with these electrodes and the other ends were connected to semiconductor parameter analyzer. Two Tygon tubes were connected to the inlet and outlet of the microfluidic channel through gold‐electroplated tubes that served as gate electrode, and the other ends were linked with syringe and pump.

### pH Sensing

Borate buffer of 10 mm at pH 8.6, 10 mm PBS buffer at pH 7.3, and 10 mm citrate buffer at pH 3.5 were prepared and injected into the microfluidic GFET biosensors at a flow rate of 50 nL min^−1^, sequentially. The incubation time lasted for 15 minutes, and the electrical responses were measured. Then the device was rinsed by Milli‐Q water (3 mL) and prepared for next incubation.

### Biomolecule Charge Type Sensing

PLL (1 mg mL^−1^) at pH 7.4 and BSA (1 mg mL^−1^) at pH 7.4 were prepared and injected into the microfluidic GFET biosensors at flow rate of 50 nL min^−1^, separately. Both of the biomolecules were incubated for 1 h at room temperature, then the device was rinsed by 1× PBS solution (3 mL). The electrical responses were measured under 1× PBS condition.

### Graphene Surface Modification

PASE of 2.5 mm dissolved in methanol was injected into the microfluidic GFET biosensor at a flow rate of 50 nL min^−1^, the incubation time was set to be 1 h at room temperature. Then the device was rinsed by 1× PBS solution (3 mL) and the electrical response was measured.

### Probe DNA Oligo and ssDNA Reporter Immobilization

Amine‐modified probe DNA oligos of 1 µm (or 2.5 µm amine‐modified ssDNA reporters) were pumped into the microfluidic GFET biosensor at flow rate of 50 nL min^−1^ and reacted with PASE for 2 h at room temperature, then followed by rinsing with 1× PBS solution (3 mL) to flush away the extra unreacted probes (or reporters). Then, the solution of 1 mm ethanolamine was used to incubate with functionalized GFET for 30 min to avoid possible nonspecific adsorption onto the graphene surface. Finally, the device was washed with 1× PBS solution (3 mL). The electrical responses of the modified and functionalized GFET were measured. The optimizations of flow rate and probe DNA concentration were also performed and the optimal conditions, 50 nL min^−1^ and 1 µm, were used respectively (Figures S13 and S14, Supporting Information).

### Nucleic Acids Hybridization Sensing

The hybridization was performed by loading complementary target (or non‐target) into the modified and functionalized microfluidic GFET biosensor at flow rate of 50 nL min^−1^ and incubated 15 min. After that, the device was rinsed by 1× PBS and the electrical responses were recorded. A comparison experiment of strand length of complementary RNA target was provided as shown in Figure S15 (Supporting Information). The sequences, namely 5′‐amine‐modified probe DNA oligo, 29‐mer complementary RNA target, 90‐mer complementary RNA target, and non‐complementary RNA target used in this work were listed in Table S1 (Supporting Information).

### Nucleic Acid Detection by Combining with CRISPR/Cas12a System

LbCas12a of 200 nm, 240 nm crRNA and 1 fm complementary target (or non‐target) were mixed and incubated at 37 °C for 30 min. Then the mixture was introduced into the modified and functionalized microfluidic GFET biosensor at flow rate of 50 nL min^−1^ and incubated 30 min. After that, the device was rinsed by 1× PBS and the electrical responses were recorded. The optimizations of CRISPR/Cas12a reaction system were also performed and the optimal conditions, 200 nm Cas12a and 240 nm crRNA, were used (Figure S16, Supporting Information). The sequences, namely 5′‐amine‐modified ssDNA reporter, crRNA, complementary and non‐complementary dsDNA targets of HAdV‐7, and ssDNA FQ reporter used in this work were listed in Table S1 (Supporting Information).

### Characterizations

Raman spectra mapping of graphene surface was characterized by LabRAM HR HORIBA with a 532 nm laser. Electrical performance responses of the microfluidic GFET biosensors were measured by FS‐Pro PRIMARIUS and B1500A KEYSIGHT.

### Statistical Analysis

Data were presented as mean ± std as specified, where *n* = 3 replicates. Statistical method to assess significance was calculated by Student's *t*‐test. * *p* < 0.05, ** *p* < 0.01, *** *p* < 0.001 were defined as statistical significances. Statistical analysis was performed using Origin 2024 and Microsoft Excel 2022.

## Conflict of Interest

The authors declare no conflict of interest.

## Author Contributions

W.Z., J.W., Z.Z. and Q.Z. conceived and planned the experiments. Q.Z. wrote the first draft of the manuscript. W.Z., J.W. and Z.Z. revised the manuscript with comments from all other authors. X.Z. provided device analysis strategies as an adviser. W.S. designed the PDMS module. Y.H., P.X. and T.Z. performed the experiments. Y.L. assisted in the Raman spectra mapping and analysis of graphene surface. C.H. contributed to the design of oligonucleotides.

## Supporting information

Supporting Information

## Data Availability

The data that support the findings of this study are available from the corresponding author upon reasonable request.
