# Supplementary material for: Modular Fabrication of Microfluidic Graphene FET for Nucleic Acids Biosensing
Source: Adv Sci (Weinh). 2024 Jul 23;11(39):2401796. doi: 10.1002/advs.202401796 (PMC11497086; doi:10.1002/advs.202401796)
Supplement: Supplementary file 1 — Supporting Information [file ADVS-11-2401796-s001.pdf]

ADVANCED SCIENCE
Open Access

# Supporting Information

for *Adv. Sci.*, DOI 10.1002/advs.202401796

Modular Fabrication of Microfluidic Graphene FET for Nucleic Acids Biosensing

*Qiongdi Zhang, Yuxuan Hao, Tonghua Zeng, Weiliang Shu, Pan Xue, Yang Li, Chi Huang, Liwei Ouyang, Xuming Zou, Zhen Zhao*, Jiahong Wang*, Xue-Feng Yu and Wenhua Zhou**

# Supporting Information

## Modular Fabrication of Microfluidic Graphene FET for Nucleic Acids Biosensing

*Qiongdi Zhang, Yuxuan Hao, Tonghua Zeng, Weiliang Shu, Pan Xue, Yang Li, Chi Huang, Liwei Ouyang, Xuming Zou, Zhen Zhao,* Jiahong Wang,* Xue-Feng Yu, and Wenhua Zhou**

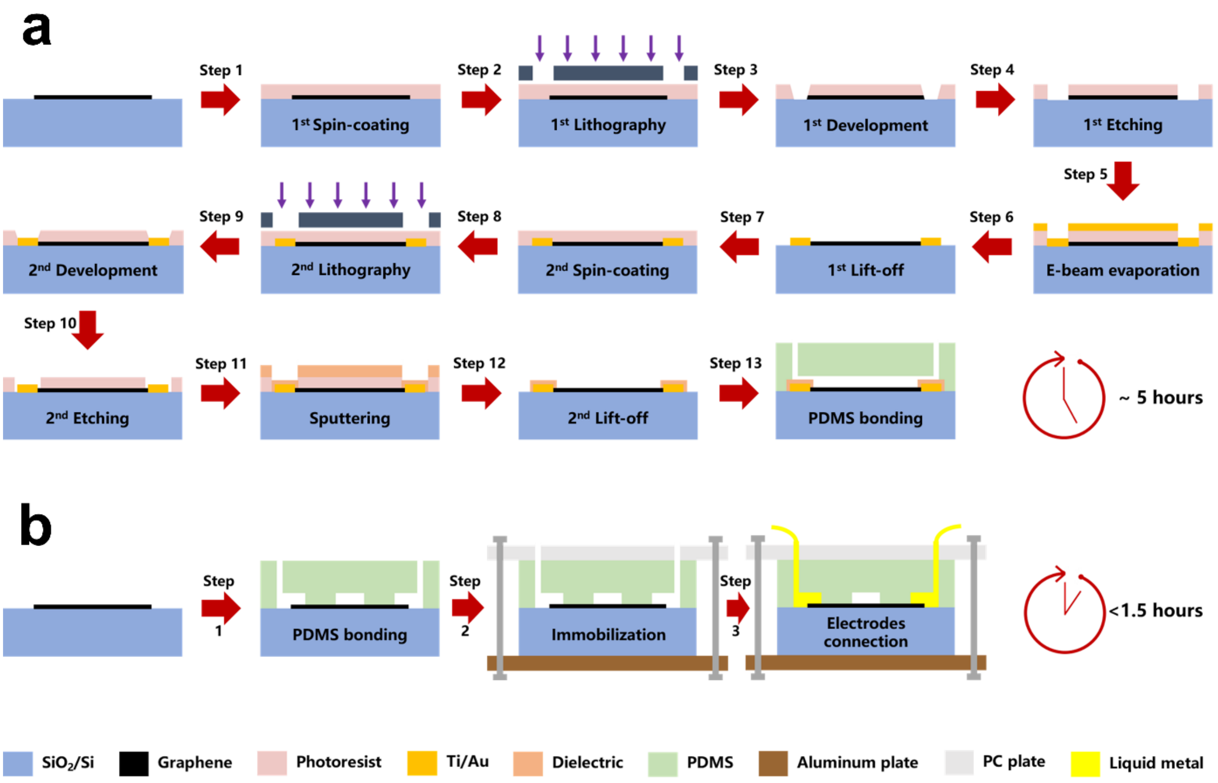


**Figure S1.** Graphical overviews of the traditional fabrication process of the microfluidic GFET biosensors (a) and our proposed modular fabrication process (b). The traditional method takes at least 13 steps and has several hours for device fabrication, while our proposed method only takes 3 steps and could be done within 1.5 hours.

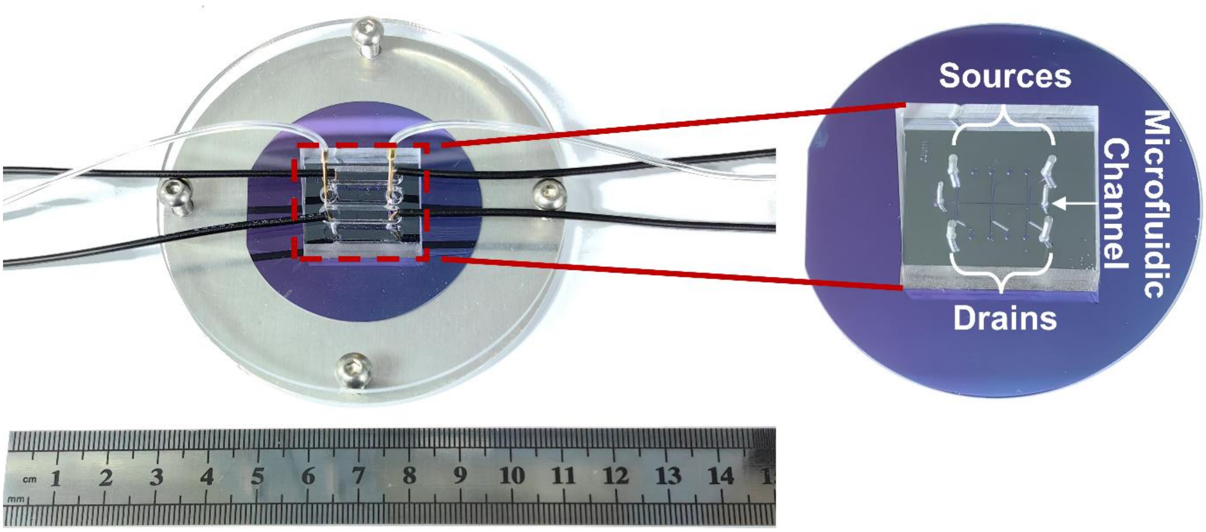


**Figure S2.** Images of the as-fabricated microfluidic GFET biosensor. A microfluidic channel is located in the center of the PDMS module with 50 μm of width, on both sides there are three pairs of source drain electrode channels that distribute symmetrically. The distance between the microfluidic channel and the electrode channel is 50 μm. The width of the electrode channel is 100 μm, illustrated in the zoomed image.

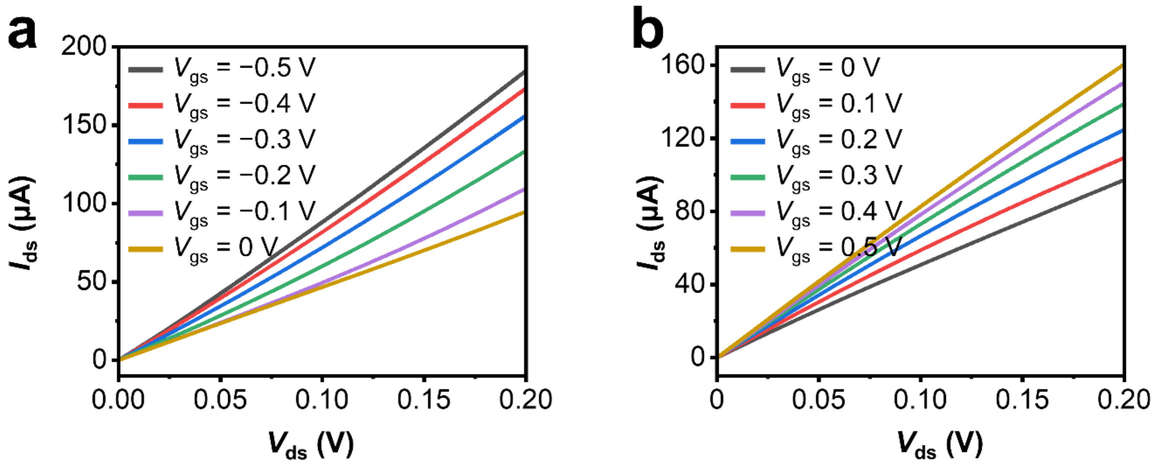


**Figure S3.** Output characteristics of the microfluidic GFET biosensor in 10× PBS solution. a) $V_{gs}$ varies from −0.5 V to 0 V. b) $V_{gs}$ varies from 0 V to 0.5 V.

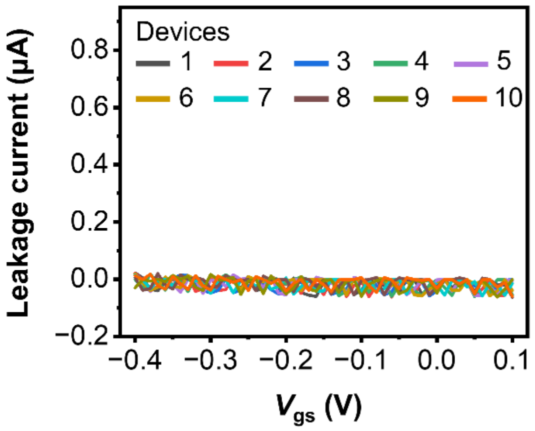


**Figure S4.** Leakage currents of 10 individual microfluidic GFET biosensors in 1× PBS solution.

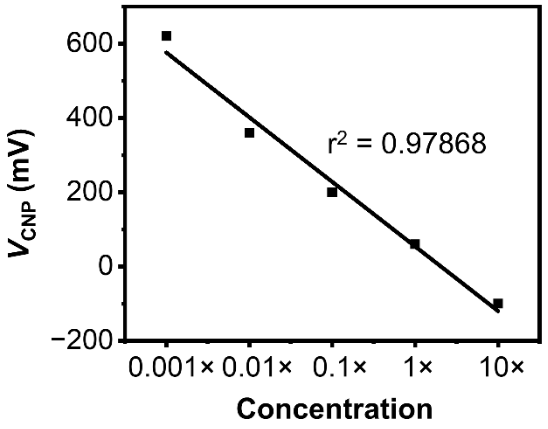


**Figure S5.** Correlation between the $V_{\mathrm{CNP}}$ of the microfluidic GFET biosensor and the PBS concentration.

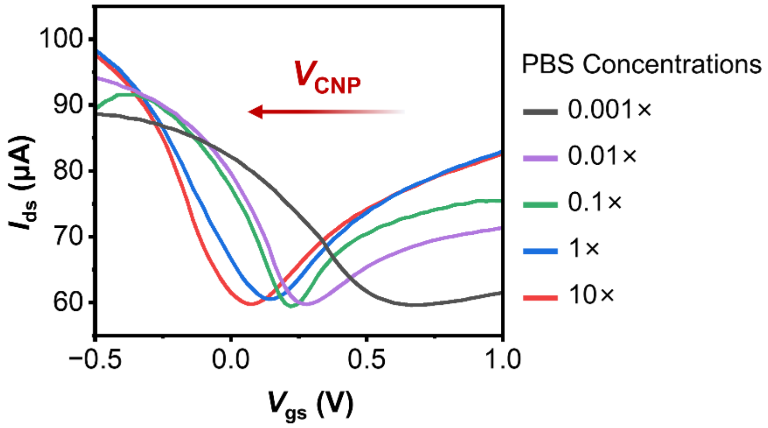


**Figure S6.** Charge transfer characteristics of the microfluidic GFET biosensor as a function of PBS concentration with channel length of 500 μm.

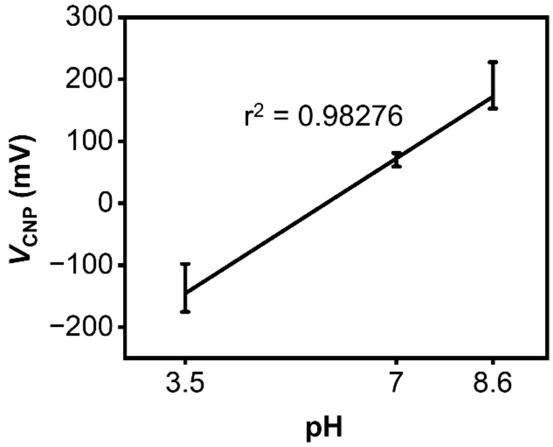


**Figure S7.** Correlation between the $V_{CNP}$ of the microfluidic GFET biosensor and the pH value, $n = 3$, mean ± std.

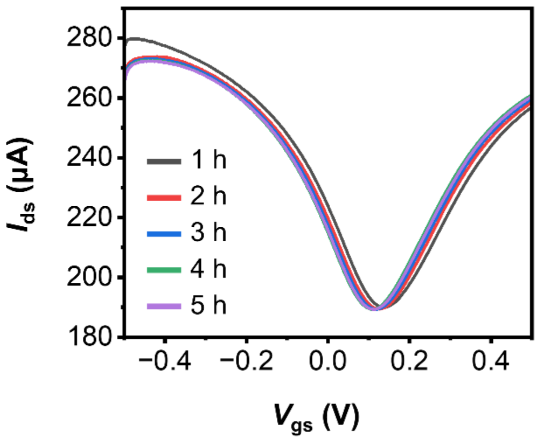


**Figure S8.** Charge transfer characteristics of the microfluidic GFET biosensor after functionalized by PASE and presented in 1× PBS buffer solution with respect to time.

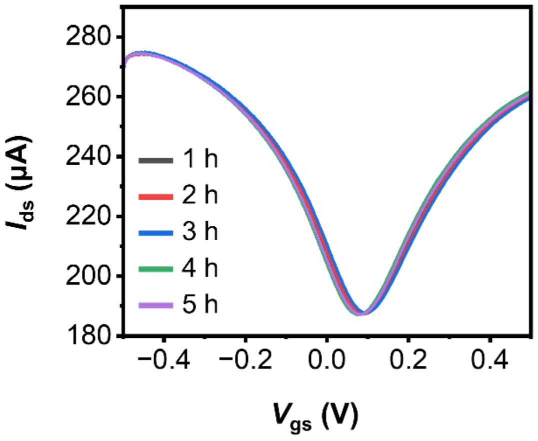


**Figure S9.** Charge transfer characteristics of the microfluidic GFET biosensor after functionalized by PASE, immobilized by probe DNA oligos, and presented in 1× PBS buffer solution with respect to time.

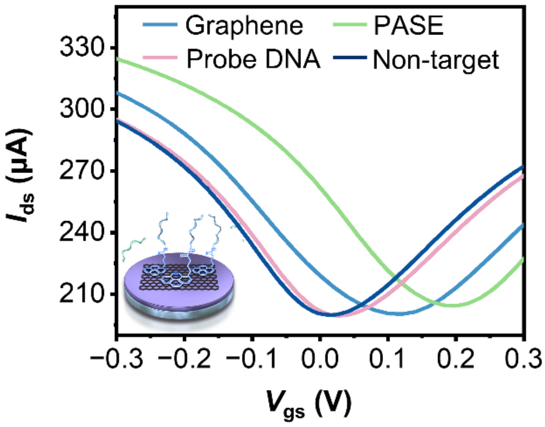


**Figure S10.** Charge transfer characteristics of step-by-step surface modification for non-target detection.

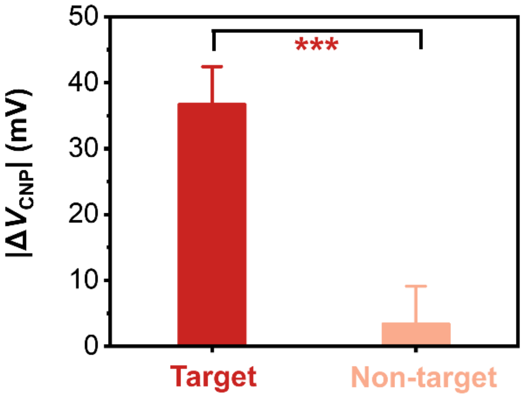

**Figure S11.** Statistical analysis of the selectivity performance of the coupled CRISPR/Cas12a-GFET biosensor for distinguishing the complementary target and non-target. $\Delta V_{CNP}$ represents the shift value of $V_{CNP}$ of the complementary target or non-target. Both detecting concentrations were 1 fM. It can be observed that the shift of the complementary target was more significant than that of the non-target, which revealed high selectivity of the proposed biosensor to target the specific nucleic acid sequence. Error bars represent mean ± std., $n = 3$. $p$ value is calculated by Student's $t$-test, *** $p < 0.001$.

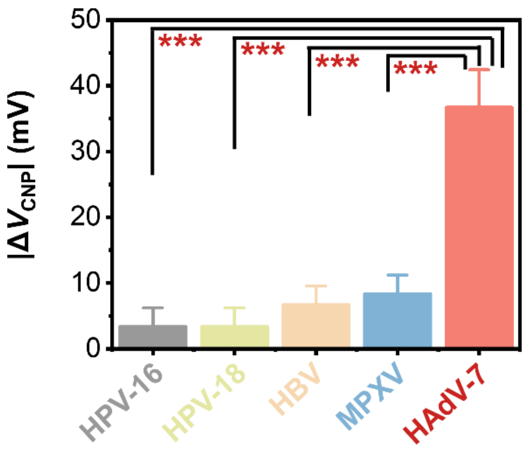


**Figure S12.** Specification of the coupled CRISPR/Cas12a-GFET biosensor for targeting the sequence of human adenovirus type 7 (HAdV-7) among several viral species. $\Delta V_{CNP}$ represents the shift value of $V_{CNP}$ of the detecting target. Both detecting concentrations were 1 fM. The results demonstrated that the proposed biosensor could provide relative high specificity. Error bars represent mean ± std., $n = 3$. $p$ values are calculated by Student's $t$-test, *** $p < 0.001$.

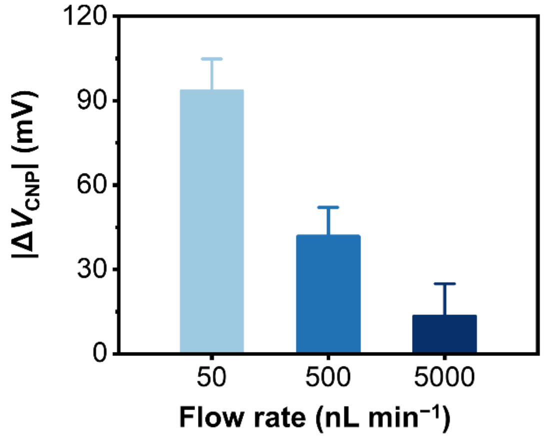


**Figure S13.** Optimization of the flow rate for nucleic acids hybridization sensing. $\Delta V_{CNP}$ represents the shift value of $V_{CNP}$ of target signal, $n = 3$, mean ± std. We compared three different

flow rates that were employed in the entire sensing process: 50 nL min$^{-1}$, 500 nL min$^{-1}$, and 5000 nL min$^{-1}$. It was found out that as the flow rate increased, the shift of $V_{CNP}$ was less obvious, resulting in a less sensitivity of the microfluidic GFET biosensor.

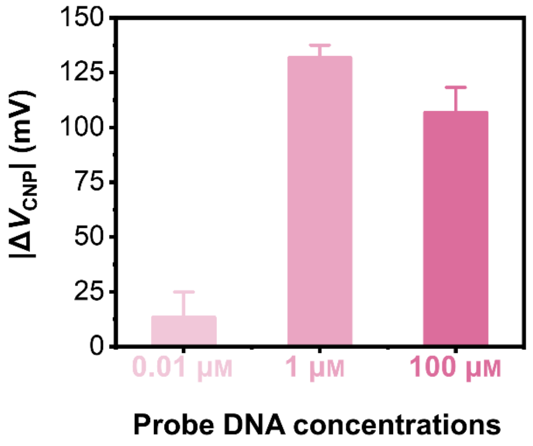


**Figure S14.** Optimization of the concentration of probe DNA oligo for nucleic acids hybridization sensing. $\Delta V_{CNP}$ represents the shift value of $V_{CNP}$ of target signal, $n$ = 3, mean ± std. We compared three different probe concentrations: 0.01 μM, 1 μM, and 100 μM. It was found out that the concentration of 1 μM produced a larger shift of signal for a given target RNA concentration in our sensing system.

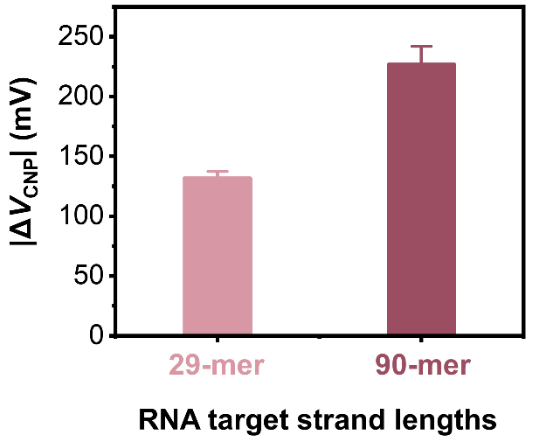


**Figure S15.** Comparison of strand length of complementary RNA target for nucleic acids hybridization sensing. $\Delta V_{CNP}$ represents the shift value of $V_{CNP}$ of RNA target signal, $n$ = 3, mean ± std. Two different strand lengths of RNA target were carried out: one was a 29-mer RNA oligo derived from the conserved nucleocapsid gene of SARS-CoV-2 genomic sequence (between 28913-28941), the other was a 90-mer RNA oligo derived from the same N gene but between 28920-29009. Both RNA oligos contained a sequence complementary to the probe

DNA. It can be observed that the 90-mer RNA target induced more significant shift of $V_{CNP}$ than the 29-mer one for a given target concentration. This phenomenon might be explained by the fact that longer target RNA oligo induced much more electrons transferring to the graphene surface, thereby resulting in a more negative shift of the $V_{CNP}$.

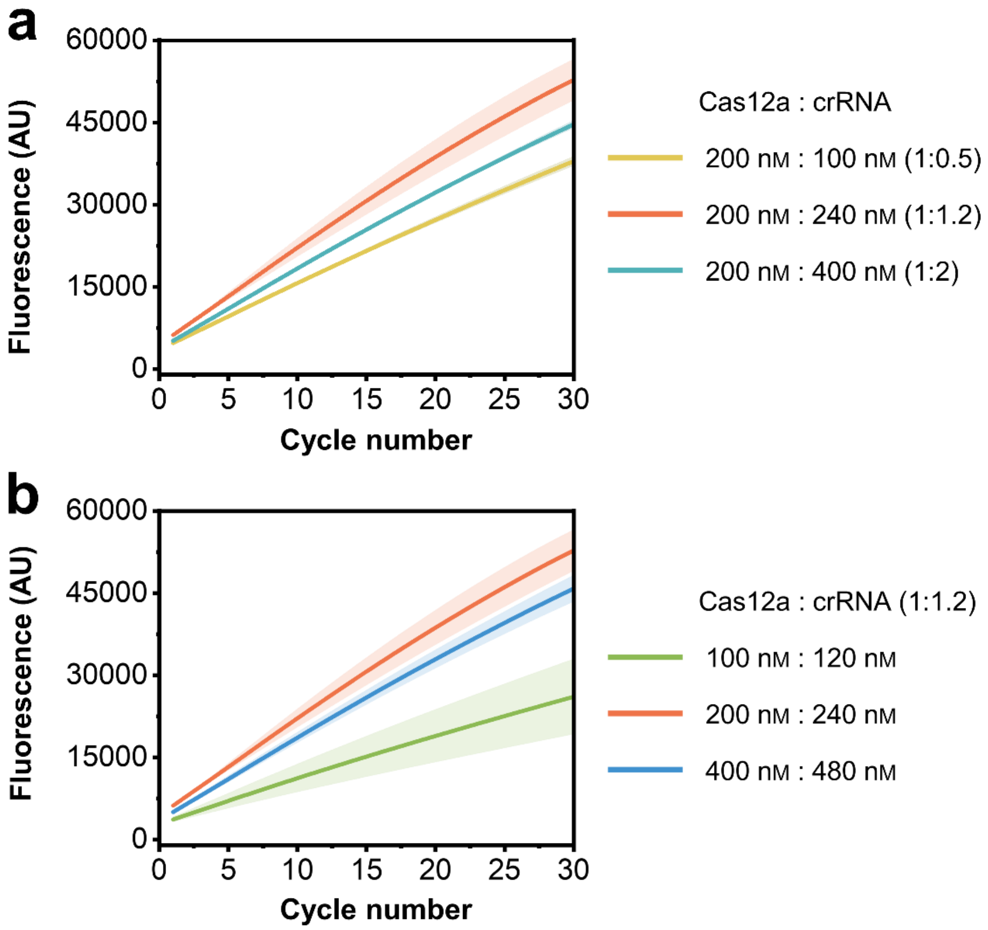


**Figure S16.** Optimizations of the trans-cleavage reaction conditions of CRISPR/Cas12a system based on fluorophore quencher (FQ)-labeled reporter assay. a) Optimization of Cas12a to crRNA ratio for a given concentration of crRNA targeting dsDNA activator. The Cas12a-crRNA complexes with different ratios (1:1, 1:1.2, 1:2) were incubated with 100 nM dsDNA activator in 1× NEBuffer™ 2 (50 mM NaCl, 10 mM Tris-HCl, 10 mM $MgCl_2$, 1 mM DTT at pH 7.9) at 37 °C for 30 min, separately. After that, 2.5 μM ssDNA FQ reporter was added into the mixture and incubated in a PCR analysis system (SLAN®-96S, Shanghai Hongshi Medical Technology Co., Ltd) at 37 °C for 30 min. The fluorescence measurements were recorded every 1 minute (ssDNA FQ reporter: $\lambda_{ex}$ = 470 nm, $\lambda_{em}$ = 510 nm). b) Optimization of the concentration of Cas12a-crRNA complexes by fixing Cas12a to crRNA ratio (1:1.2) and the concentration of dsDNA activator (100 nM). Three concentrations were compared by carrying

out the same process as mentioned above. Error bars represent mean ± std., where $n$ = 3 replicates.

**Table S1.** The sequences of DNA and RNA used in this work.

| DNA/RNA | Sequence (5'-3') |
|---|---|
| 5'-amine-modified probe DNA oligo | $H_2N$ - $C_6$ - GCAGATTTCTTAGTGACAGTTTGGCCTTG |
| 29-mer complementary RNA target of N gene of SARS-CoV-2 genomic sequence (between 28913-28941) | CAAGGCCAAACUGUCACUAAGAAAUCUGC |
| 90-mer complementary RNA target of N gene of SARS-CoV-2 genomic sequence (between 28920-29009) | CUGCUUGACAGAUUGAACCAGCUUGAGAGCAAAAUGUCUGGUAAAGGCCAAC AACAACAAGGCCAAACUGUCACUAAGAAAUCUGCUGCU |
| Non-complementary RNA target | CAAGGCCAAACUGUCACUAAGGAAUCUGC |
| 5'-amine-modified ssDNA reporter | $H_2N$ - $C_6$ - CTCTCATTTTTTTTTTAGAGAG |
| crRNA | UAAUUUCUACUAAGUGUAGAUGAGUCAUGCGCAUGUAAAAC |
| Complementary dsDNA target of conserved hexon gene of HAdV-7 (between 11703-11761) | GAGGTAAAGATAGATGGGTTTTACATGCGCATGACTCTAAAGGTACTAACTCTA AGCGA |
| Non-complementary dsDNA target of conserved hexon gene of HAdV-7 (between 12994-13052) | AACAGAGCGTTGGACTGTTTCTGATGCAGGAGGGGGCGACACCTACCGCCGC GCTGGAC |
| ssDNA FQ reporter | FAM - TCCCCCCT - TAMRA |

**Table S2.** Comparison between the proposed microfluidic GFET nucleic acids biosensor and other reported studies.

| Electrode lithography required | Passivation lithography required | Method accessible | Processing after graphene transferred | Limit of detection (LOD) | Probe used | Microfluidic channel used | Reference |
|---|---|---|---|---|---|---|---|
| Yes | Yes | No | Yes, passivation lithography | 25 aM | DNA | No | [1] |
| Yes | No, but with 0.2 μA gate leakage | No | No | 10 fM | PNA | No | [2] |

| | | | | | | | |
|---|---|---|---|---|---|---|---|
| Yes | No | No | Yes, electrode lithography | 100 fM | DNA | Yes | [3] |
| Yes | Yes | No | Yes, all steps | 100 fM | DNA | Yes | [4] |
| No | No | Yes | No | 1 pM | DNA | No | [5] |
| No | No | Yes | No | 1 pM | DNA | No | [6] |
| Yes | Yes | No | Yes, all steps | 10 pM | DNA | Yes | [7] |
| Yes | No | No | No | 1 nM | DNA | Yes | [8] |
| No | No | Yes | No | 10 fM | DNA | Yes | **This work** |

References

[1] R. Campos, J. Borme, J. R. Guerreiro, G. Machado, Jr. M. F. Cerqueira, D. Y. Petrovykh, P. Alpuim, *ACS Sens.* **2019**, *4*, 286.

[2] C. Zheng, L. Huang, H. Zhang, Z. Sun, Z. Zhang, G.-J. Zhang, *ACS Appl. Mater. Interfaces* **2015**, *7*, 16953.

[3] S. Xu, S. Jiang, C. Zhang, W. Yue, Y. Zou, G. Wang, H. Liu, X. Zhang, M. Li, Z. Zhu, J. Wang, *Appl. Surf. Sci.* **2018**, *427*, 1114.

[4] G. Xu, J. Abbott, L. Qin, K. Y. M. Yeung, Y. Song, H. Yoon, J. Kong, D. Ham, *Nat. Commun.* **2014**, *5*, 4866.

[5] J. Sun, X. Xie, K. Xie, S. Xu, S. Jiang, J. Ren, Y. Zhao, H. Xu, J. Wang, W. Yue, *Nanoscale Res. Lett.* **2019**, *14*, 248.

[6] T. Y. Chen, P. T. Loan, C. L. Hsu, Y. H. Lee, J. Tse-Wei Wang, K. H. Wei, C. T. Lin, L. J. Li, *Biosens. Bioelectron.* **2013**, *41*, 103.

[7] S. Xu, J. Zhan, B. Man, S. Jiang, W. Yue, S. Gao, C. Guo, H. Liu, Z. Li, J. Wang, Y. Zhou, *Nat. Commun.* **2017**, *8*, 14902.

[8] H. E. Kim, A. Schuck, J. H. Lee, Y.-S. Kim, *Sens. Actuators B Chem.* **2019**, *291*, 96.
